# Bovine Lactoferrin Protects Dextran Sulfate Sodium Salt Mice Against Inflammation and Impairment of Colonic Epithelial Barrier by Regulating Gut Microbial Structure and Metabolites

**DOI:** 10.3389/fnut.2021.660598

**Published:** 2021-04-16

**Authors:** Shalong Wang, Jingyu Zhou, Da Xiao, Guoshun Shu, Li Gu

**Affiliations:** ^1^Department of General Surgery, The Second Xiangya Hospital of Central South University, Changsha, China; ^2^Department of General Surgery, Shekou People's Hospital of Central South University, Shenzhen, China; ^3^Department of Gastroenterology, The Second Xiangya Hospital of Central South University, Changsha, China

**Keywords:** colitis, gut microbiota, intestinal epithelial barrier, gut microbial, bovine lactoferrin

## Abstract

**Background:** Ulcerative colitis is characterized by relapsing and remitting mucosal inflammation. Bovine lactoferrin (BL) is a multifunctional protein that could regulate the intestinal flora and has anti-inflammatory effects. The aim of this study was to investigate the therapeutic effect of BL on colitis.

**Methods:** Dextran sulfate sodium salt (DSS) was utilized to establish a mouse model of colitis. BL was administered to treat DSS mice. The weight, the activity, and fecal status of the mice were recorded every day. Disease activity index was calculated. After the mice were euthanized, the colon length was measured. Hematoxylin and eosin staining was used to observe the pathological changes of the colon, and histological activity index was calculated. The myeloperoxidase (MPO) activity of colon tissue was measured. Western blot and immunohistochemistry were used to detect the expressions of Claudin-1, Occludin, and ZO-1. The expressions of IL-1β, IL-6, IL-10, TNF-α, and TGF-β in colon tissue were detected by ELISA. The protein expressions of MUC2, Reg3γ, β-defensin (HBD-2), and cAMP were detected by immunofluorescence (IF). 16S rDNA sequencing determined the type and structure of intestinal flora. Liquid chromatography–tandem mass spectrometry (LC-MS/MS) measured the metabolites of the intestinal flora.

**Results:** Compared with the DSS group, the mice's weight in the BL group was higher and the length of the colon was longer. At the 14th day, MPO activity was higher in the BL group. The expressions of Claudin-1, Occludin, and ZO-1 in the colon were up-regulated in the BL group compared with the DSS group. The expressions of IL-1β, IL-6, and TNF-α were lower. The expressions of IL-10 and TGF-β were higher. IF showed that the expressions of MUC2 and β-defensin (HBD-2) were down-regulated, and the expressions of Reg3γ and cAMP were up-regulated. The 16S rDNA sequencing results showed that the alpha diversity and beta diversity were notably changed in the DSS mice treated with BL. Metabolomics results showed that BL changed purine metabolism in the DSS mice.

**Conclusion:** BL alleviated colitis in mice by improving the inflammatory response and the structure of the colon barrier in the colon. BL changed the composition and metabolites of the intestinal flora. Thus, BL might be an effective nutritional supplement for colitis treatment.

## Backgrounds

The multifactorial pathophysiology of UC includes genetic predisposition, epithelial barrier defects, dysregulated immune responses, microbial dysbiosis, and environmental factors. Colitis could cause frequent abdominal pain, diarrhea, and even colon cancer (1, 2). But most of the drugs used in colitis, such as 5-aminosalicylic acid (5-ASA) or corticosteroids, would interfere with the patient's metabolism and would cause side effects. Therefore, we need to seek a safer and better drug for colitis patients.

Colitis is caused by inflammation of the intestinal tissue and destroys the intestinal barrier. The dextran sulfate sodium salt (DSS) colitis model is the most widely used inflammatory enteritis model (3). In DSS colitis models, the intestinal mucosa and epithelial cells were destroyed and inflammatory cells were activated (4). In addition, the lack of adaptive immunity in the intestines made bacteria and monocytes enter the intestinal mucosa, leading to an intestinal barrier imbalance (5). Therefore, it is crucial to maintain a stable microenvironment in the intestines. Many proteins regulate intestinal homeostasis, such as Claudin-1, Occludin, and ZO-1. Claudin-1 is involved in the regulation of intestinal epithelial barrier homeostasis by regulating Notch signal (6). In the area of damaged intestinal, the expression of Claudin-1 is up-regulated (7). Occludin is a transmembrane junction protein, and its C-terminus could directly interact with tight junction protein ZO-1 (8). By regulating CASP3 transcription and Caspase-3 expression, ZO-1 could regulate epithelial cell apoptosis and survival (9).

Intestinal inflammation is related to the imbalance of intestinal flora (10). The metabolites of intestinal flora affected the host's immune homeostasis, normal metabolism, and the integrity of the mucosa (11). Most studies have proved that fecal microbiota transplantation (FMT) could help colitis patients in recovering better (12). Intestinal microorganisms might produce abundant metabolites, among which butyrate could provide energy to colon cells, maintain the integrity of colon mucosa, and have anti-inflammatory and anticancer effects (13).

Bovine lactoferrin (BL) is a non-heme iron-binding glycoprotein (14), which promotes the proliferation and differentiation of intestinal epithelial cells (15). Besides, BL also has antibacterial and antiviral properties (16, 17). Studies have shown that in the early stage of antiviral treatment, BL may prevent the virus from entering colon host cells (18). Thus, BL is a natural immune molecule. Moreover, BL regulated the synthesis of ferroportin through down-regulation of IL-6 and up-regulated anemia in pregnant women (19). These results indicate that BL could play a therapeutic role by improving inflammation. However, there are few studies on the treatment of colitis with BL

Therefore, our study aims to prove the effect of BL on the inflammation and intestinal barrier of the DSS mice. We also intend to explore the effect of BL on the structure of intestinal flora and its metabolites in colitis and provide a new idea for the treatment of colitis.

## Methods

### Dextran Sulfate Sodium Salt Model

Thirty-six mice were purchased from Animal Experiment Center of Xiangya Medical College, Central South University. All mice were fed for 7 days to adapt to the environment before the experiment. Colitis mouse model was induced using DSS. All mice were randomly divided into three groups, with 12 mice in each group. The control group drank water normally during the experiment. In the model group (DSS) group, the mice drank 4% DSS solution (20) freely for 7 days and then were fed with normal saline for 14 days. In the DSS+BL group, 4% DSS solution was drunk freely for 7 days, and then BL (100 mg/kg) (21) was gavaged for 14 days. Seven days after intragastric administration of DSS, the mice's weight was recorded every day, and the disease activity index (DAI) was calculated according to the weight and defecation of the mice for 14 consecutive days. After 14 days, the mice were sacrificed, and the length of their colon was measured. All experimental protocols were approved by Animal Ethics of the Second Xiangya Hospital, Central South University (2020844). The care and handling of animals comply with the guidelines of the National Institutes of Health.

### Determination of Disease Activity Index

By measuring the DAI, the health of the rats was evaluated. We calculate the total weight loss, diarrhea, and blood in the stool from the first day as the clinical disease score. Grading rules are as follows: 0 points, mice weight changes within 1%, stool shape normal, and no rectal bleeding; 1 point, mice lost 1–5% weight, and stool became softer with weak hemoccult; 2 points, mice lost 5–10% weight, accompanied with moderate diarrhea and blood in the stool; 3 points, mice lost 10–15% weight, accompanied with diarrhea and fresh rectal bleeding; and 4 points, mice lost more than 15% of body weight, accompanied with severe bloody stools.

### H&E Staining

The mice were sacrificed 14 days later, and colon tissue was taken. Leica microtome sliced the embedded tissue at 15 μm, following the steps in the H&E kit (Wellbio, China) instructions for staining. According to the results of H&E staining, goblet cells, inflammatory cells, and crypts in colon tissue were calculated to determine histological activity index (HAI). The histological activity scoring rules are as follows: (1) intestinal epithelial injury [no injury (0 points), massive loss of goblet cells (1 point), small number of crypts + massive loss of goblet cells (3 points), and large numbers of crypts absent (4 points)]; and (2) inflammatory cell infiltration [no inflammatory cell infiltrated (0 points), inflammatory cell infiltrated around the crypt (1 point), inflammatory cell infiltrated into the muscularis mucosa (2 points), inflammatory cell infiltrated to the muscularis mucosa with edema (3 points), and inflammatory cells infiltrated the submucosa (4 points)]. The scores of the above two items are added to get the HAI score (0–8 points).

### Immunohistochemistry

The slices were dewaxed in water and then placed in xylene for 20 min, which were performed three times. After that, the sections were placed in 100, 95, 85, and 75% ethanol for 5 min. Slices were soaked in distilled water for 5 min. The slices were immersed in 0.01 M of citrate buffer (pH 6.0) and boiled for 20 min. After being cooled to room temperature, the slices were washed with 0.01 M of PBS (pH 7.2~7.6) for 3 min, which were performed three times. Then 1% periodic acid was added, and the slices were placed at room temperature for 10 min. Slices were washed with PBS for 3 min, which were performed three times. Diluted primary antibodies Claudin-1 (1:100, rabbit, 13050-1-AP, PTG), Occludin (1:100, rabbit, 13050-1-AP, PTG), and ZO-1 (1:100, rabbit, 13050-1-AP, PTG) were added to the slices and put at 4°C overnight. Pan secondary antibody was added, and slices were incubated at 37°C for 30 min. DAB (Nakasugi Golden Bridge) was added to dye slices for 5–10 min. Hematoxylin (Wellbio, China) was used to dye cell nuclei for 5–10 min, and then they were washed with distilled water. They were dehydrated in all levels of alcohol (60–100%) and transparent in xylene. The slides were mounted with neutral gum (Sigma) and then observed.

### Immunofluorescence

We deparaffinized the sections to water and placed in xylene for 20 min, which were performed three times. Slices were put in 100, 95, 85, and 75% ethanol in sequence for 5 min at each level. Slices were washed with distilled water for 5 min. The slices were immersed in citrate buffer (pH 6.0) and boiled in an electric furnace or microwave oven. After being cooled, slices were washed with 0.01 M of PBS (pH 7.2 ~ 7.6) for 3 min, which were performed three times. Slices were place in sodium borohydride solution at room temperature for 30 min. The sections were placed in Sudan black dye solution at room temperature for 5 min. Slices were blocked with 10% normal serum/5% bovine serum albumin (BSA) for 60 min. Slices were placed in appropriate first antibody, cAMP (1:50, rabbit, ab76238, Abcam, UK), MUC2 (1:50, rabbit, ab76774, Abcam, UK), Reg3γ (1:50, rabbit, ab233480, Abcam, UK), β-defensin (HBD-2) (1:50, rabbit, bs-1296r, Bioss, China), Claudin-1 (1:50, rabbit, 13050-1-AP, PTG), and ZO-1 (1:50, rabbit, 21773-1-AP, PTG), overnight at 4°C. Slices were incubated with CoraLite488-conjugated Affinipure Goat Anti-Rabbit IgG(H+L) (SA00013-2, Proteintech, USA) and incubated at 37°C for 90 min. Slices were stained in the nucleus with DAPI (Wellbio, China) working solution at 37°C for 10 min. Slices were stored in the dark or observed under a fluorescence microscope.

### Western Blot

After the colon tissue was taken out, 200 μl of radioimmunoprecipitation assay (RIPA), protease inhibitor mixture was added, and the tissue sample was broken by ultrasonic for 1.5 min and lysis on ice for 10 min. The supernatant was collected after centrifugation at 4°C and 12,000 rpm for 15 min. Bicinchoninic acid (BCA) protein quantification kit was used to quantitatively analyze the protein. In the protein supernatant, 5× loading buffer was added, mixed well, boiled for 7 min, and places in an ice box for quick cooling. Twenty micrograms of protein sample was added into 10% separating gel and 4.8% concentrated gel, and electrophoresis was performed. Protein was transferred to polyvinylidene difluoride (PVDF) membrane. After being blocked with 5% milk at room temperature for 2 h, the protein band was incubated with the primary antibody overnight at 4°C. The primary antibodies were Claudin-1 (1:2,000, rabbit, ab211737, Abcam, UK), Occludin (1:2,000, rabbit, ab45171, Abcam, UK), ZO-1 (1:2,000, rabbit, 21773-1-AP, Proteintech, USA), MUC2 (1:500, rabbit, ab76774, Abcam, UK), Reg3γ (1:500, rabbit, ab233480, Abcam, UK), HBD-2 (1:500, rabbit, bs-1296r, Bioss, China), cAMP (1:500, rabbit, ab76238, Abcam, UK), and β-actin (1:5,000, rabbit, 60008-1-Ig, Proteintech, USA). The antigen species of the primary antibody was incubated with a suitable secondary antibody at room temperature for 2 h and then developed with enhanced chemiluminescence (ECL) solution kit. The band pictures were analyzed with ImageJ to obtain protein expression data.

### Enzyme-Linked Immunosorbent Assay

The tissues were added with 200 μl of RIPA and protease inhibitor and sonicated for 1.5 min. The supernatant was obtained from centrifuging the tissue homogenate at 4°C and 12,000 rpm for 20 min. According to the instructions of the ELISA kit, by measuring the optical density (OD) value with the microplate reader, the concentration of IL-1β (CSB-E08054m, Wuhan Huamei Biological Engineering Co., Ltd., China), IL-6 (CSB-E04639m, Wuhan Huamei Biological Engineering Co., Ltd., China), IL-10 (CSB-E04594m, Wuhan Huamei Biological Engineering Co., Ltd., China), TNF-α (CSB-E04741m, Wuhan Huamei Biological Engineering Co., Ltd., China), and TGF-β1 (CSB-E04726m, Wuhan Huamei Biological Engineering Co., Ltd., China) factors was calculated.

### Myeloperoxidase Activity Measurement

According to the instructions, the peroxidase activity in rat colon tissue was determined by the guaiacol colorimetric method. Myeloperoxidase (MPO) activity could be calculated by comparing tissue OD value with A value. The unit of enzyme activity is the number of micromoles of xylophenol oxidized by the enzyme contained in a 1-g sample within 1 min.

### Intestinal Flora Metabolomics

The intestinal excrement of mice was placed into 1.5-ml centrifuge tubes. There were eight sample replicates in each group. Liquid nitrogen was added to the centrifuge tube and weighed. According to the weight, nine times of the volume of the internal standard substance containing ^13^C stable isotope was added with the pre-cooled extract liquid and mixed. Then, samples were left on ice for 5–10 min. Samples were in high-speed low-temperature centrifugation for 10 min at 4°C in 16,000 rpm. The supernatant was taken, and liquid chromatography–tandem mass spectrometry (LC-MS/MS) analysis was utilized. After obtaining the original data, we analyzed and sorted the data.

### 16S rDNA Sequencing

DNA was extracted from a single mouse stool sample by repeated beading and column purification methods. DNA quality was checked by agarose gel and quantified by Quant-iT dsDNA analysis kit (Cat.12640ES76, Shanghai Yisheng Biological Technology Co., Ltd.). The DNA was subjected to MiSeq sequencing (Illumina) according to the 2 × 300 pair termination protocol. The V4–V5 hypervariable region (primer sets 515F and 806R) of the 16S rDNA gene was amplified and sequenced using the method/manual of the manufacturer. After the raw data were processed, each species' operational taxonomic units (OTUs) were obtained, and species annotations were made for each OTU. According to the obtained species information and based on the species' abundance distribution, the final results were plotted.

### Data Analysis

The data were analyzed using GraphPad Prism 7.0 (GraphPad Software Inc., San Diego, CA). All data were expressed as mean ± standard deviation. We used one-way ANOVA for data analysis between multiple groups. *P* < 0.05 was statistically significant. We utilized Kruskal–Wallis test (between multiple groups) and Wilcoxon test (between two groups) to analyze the relative abundance of species. Principal component analysis (PCA) and principal coordinate analysis (PCoA) were performed using the Anosim analysis, Adonis analysis, and analysis of differences in bacterial species abundance based on the Wald test method. The Spearman analysis was used to analyze the correlation between different microorganisms and different metabolites.

## Results

### Bovine Lactoferrin Alleviated Dextran Sulfate Sodium Salt-Induced Colitis in Mice

**Figure 1:**
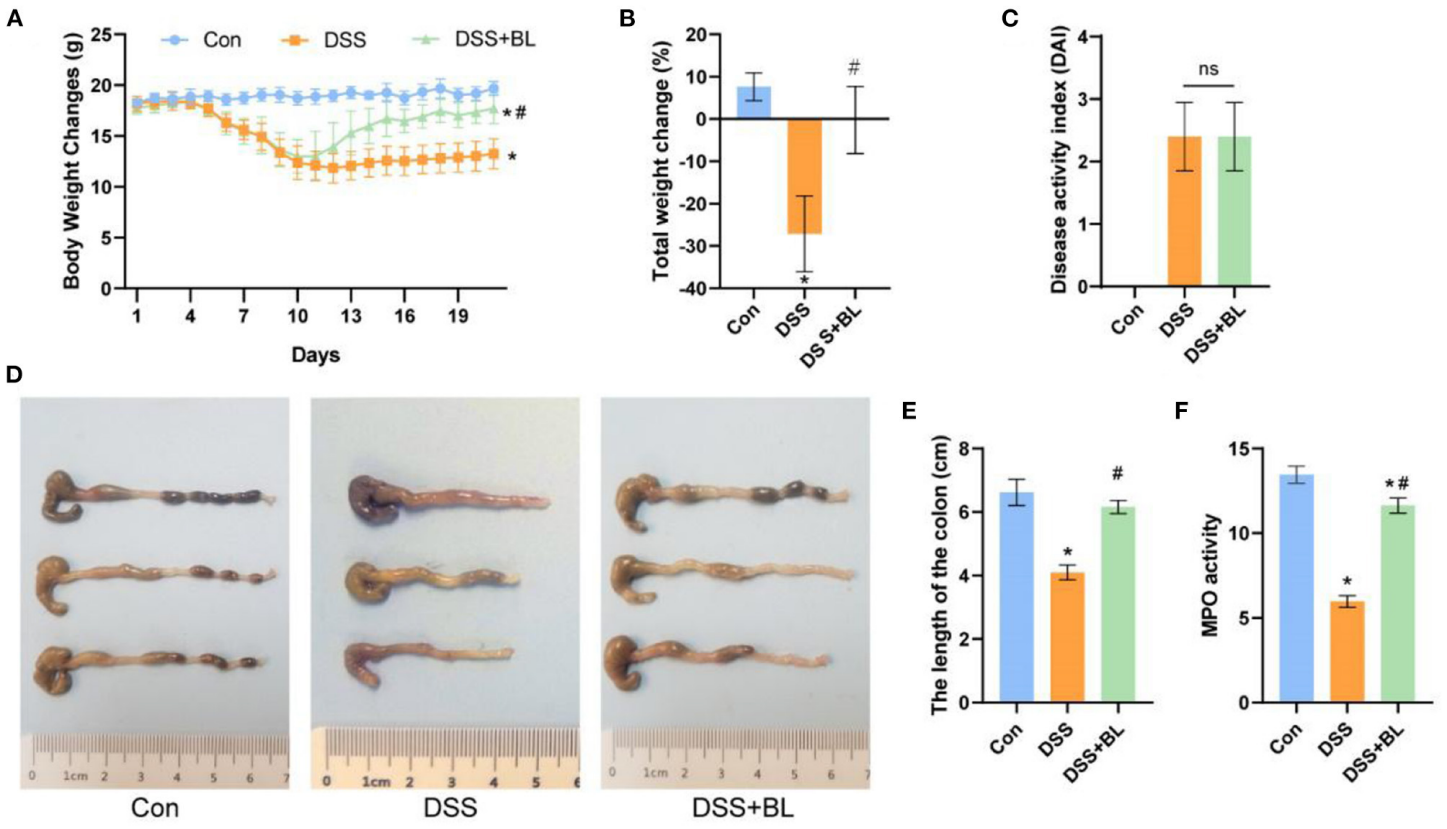
Effects of bovine lactoferrin (BL) on changes in body weight, colon morphology, and myeloperoxidase (MPO) activity in dextran sulfate sodium salt (DSS) mice. **(A)** Changes of body weight of mice in each group within 19 days since start of modeling. **(B)** Changes of the rate of total weight of mice in each group within 19 days. **(C)** Changes of disease activity index (DAI) index on the seventh day after modeling. **(D)** Pictures of colons in each group. **(E)** Changes of colon length of mice in each group. **(F)** Changes of MPO activity in colon of mice in each group. One-way ANOVA was used to compare the three groups. *P < 0.05 vs. control; ^#^*P* < 0.05 vs. DSS. *n* = 6.

In order to explore the therapeutic effect of BL on the DSS mice, we tested the changes in the overall health of the mice during the administration period after modeling. We found that on the seventh day after modeling, the weight of the DSS mice decreased significantly, and the DAI score of two DSS groups was higher than that of the control group (*P* < 0.05), indicating that the colitis models were successfully created. After the 10th day, compared with that of the DSS group, the weight of the DSS+BL group increased gradually (Figure 1A). Compared with the first day, the weight of the control group increased by 8%, the weight of the DSS group decreased by 26% (*P* < 0.01), and the weight of the DSS+BL group changed little (Figure 1B). On the 14th day, we calculated the DAI based on the overall weight change and defecation of the mice. The DAI scores of the control group were the lowest. The scores of the DSS group and DSS+BL group increased, compared with the control group (Figure 1C). But the DAI between the two DSS groups had no significant difference. Meanwhile, we found that the length of the colon in the DSS group had been shortened to 4 cm, while the colon length of the control group and the DSS+BL group remained around 6 cm (Figures 1D,E; *P* < 0.05). The MPO activity was measured, and we found that the peroxidase activity in the DSS+BL group was up-regulated, compared with the DSS group (Figure 1F; *P* < 0.05). Therefore, these results suggested that BL reduced the related symptoms of the DSS mice.

### Effects of Bovine Lactoferrin on Intestinal Epithelial Barrier in Dextran Sulfate Sodium Salt Mice

**Figure 2:**
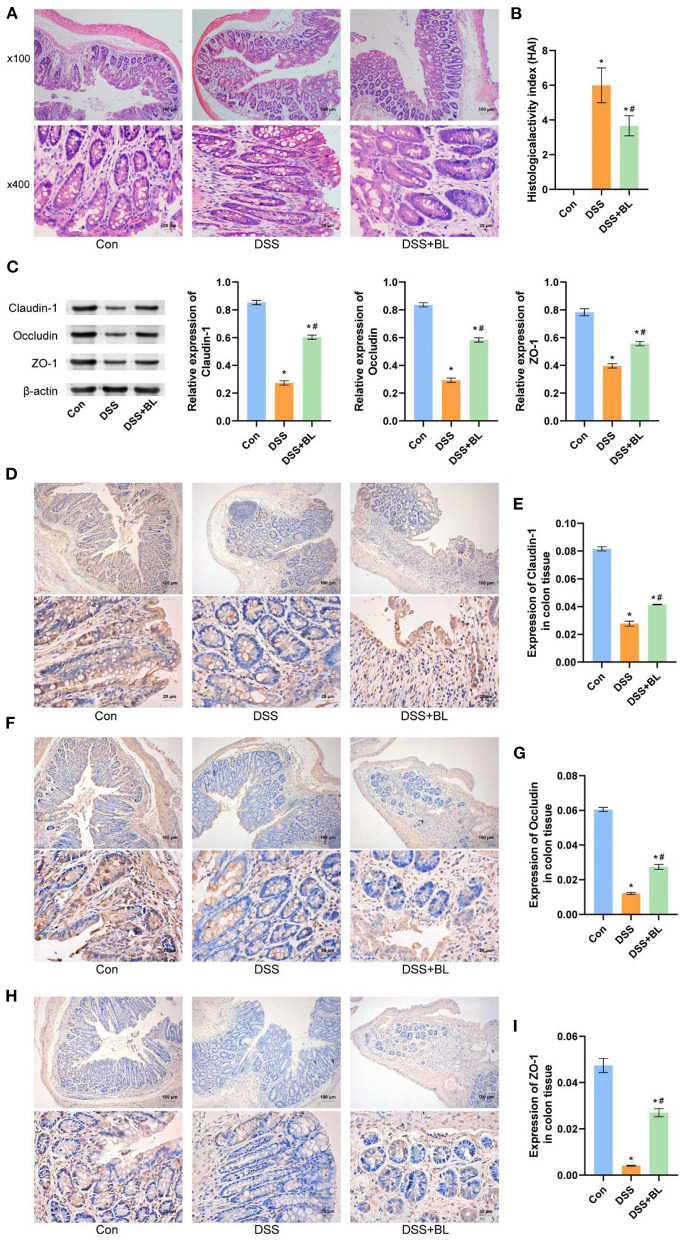
Effects of bovine lactoferrin (BL) on colon tissue morphology and expression of colonic barrier-related connexin proteins in dextran sulfate sodium salt (DSS) mice. **(A)** H&E staining of colon tissue in mice. **(B)** Changes of histological activity index of colon tissue in mice. **(C)** Western blot results and analysis of Claudin-1, Occludin, and ZO-1. **(D,E)** Immunohistochemistry results and analysis of Claudin-1. **(F,G)** Immunohistochemistry results and analysis of Occludin. **(H,I)** Immunohistochemistry results and analysis of ZO-1. One-way ANOVA was used to compare the three groups. *P < 0.05 vs. control; ^#^*P* < 0.05 vs. DSS. *n* = 6.

In order to explore the effect of BL on the intestinal epithelial barrier of the DSS mice, we utilized H&E staining to detect the pathological changes of colon tissue (Figure 2A). In the DSS groups, lymphocytes infiltration was increased and gathered into the crypts, epithelial cells were damaged, and goblet cells were markedly decreased, as compared with those in the DSS+BL groups (*P* < 0.01). We counted the goblet cells, inflammatory cells, and crypts in colon tissue and calculated the HAI. The HAI results showed that the HAI index of the DSS+BL groups was significantly lower than that of the DSS groups (Figure 2B; *P* < 0.05). Then, we performed Western blot (WB) and immunohistochemistry (IHC) to detect the level of the colonic barrier-related connexin proteins Claudin-1, Occludin, and ZO-1. WB (Figure 2C) and IHC (Figures 2D–I) results showed that the level of three proteins was the lowest in the DSS group. In the DSS+BL group, the expression of the three proteins enhanced than the DSS group and tended to the normal level (*P* < 0.05). These results indicated that BL could help damage colonic epithelial barrier recovery in the DSS mice.

### Effects of Bovine Lactoferrin on Intestinal Inflammation and Colonic Mucosa in Dextran Sulfate Sodium Salt Mice

**Figure 3:**
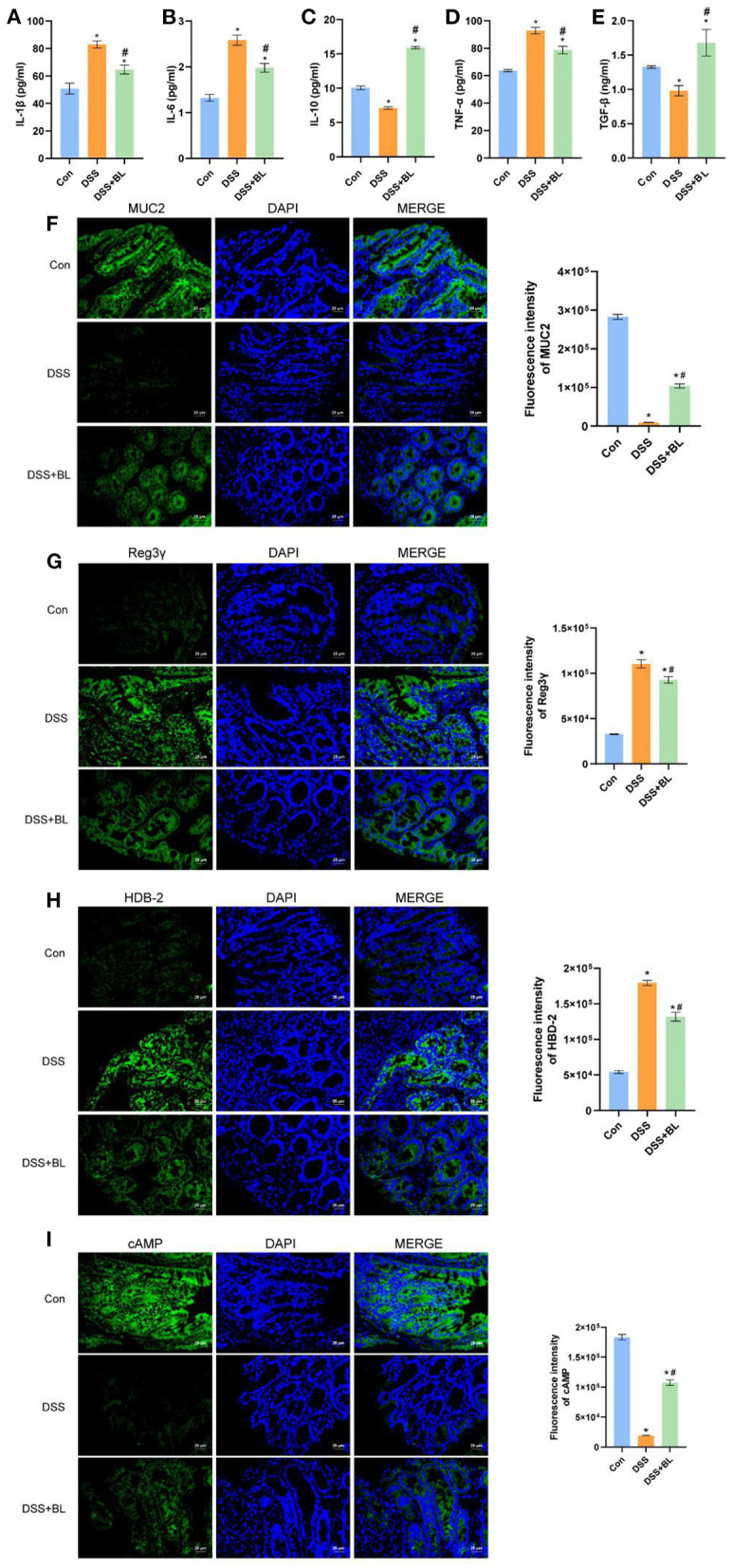
Effects of bovine lactoferrin (BL) on inflammatory factors and expression of colonic mucosa-related defense proteins in dextran sulfate sodium salt (DSS) mice. **(A–E)** ELISA of inflammatory factors, IL-1β, IL-6, IL-10, TGF-α, and TNF-β of colon tissue in mice. **(F)** Immunofluorescence results and analysis of MUC2. **(G)** Immunofluorescence results and analysis of Reg3γ. **(H)** Immunofluorescence results and analysis of HBD-2. **(I)** Immunofluorescence results and analysis of cAMP. One-way ANOVA was used to compare the three groups. *P < 0.05 vs. control; ^#^*P* < 0.05 vs. DSS.

In order to test the effect of BL on colon inflammation in the DSS mice, we used ELISA to examine the expression of related inflammatory factors or anti-inflammatory factors in the colon. The ELISA results showed that, compared with those of the DSS group, the levels of inflammatory factors IL-1β, IL-6, and TNF-α (Figures 3A,B,D) were down-regulated in the DSS+BL groups, and the expression levels of anti-inflammatory factor IL-10 and TGF-β (Figures 3C,E) were up-regulated (*P* < 0.05). We used immunofluorescence (IF) to assess the expressions of colonic mucosa-related defense proteins, MUC2, Reg3γ, β-defensin (HBD-2), and cAMP. Compared with those in the DSS group, the expressions of MUC2 (Figure 3F) and cAMP (Figure 3I) significantly ascended in the DSS+BL groups (*P* < 0.05), while the expressions of Reg3γ (Figure 3G) and β-defensin (HBD-2) (Figure 3H) were inhibited. These results shown that BL might decrease the inflammation and promote the repair of colonic mucosa in the colon of the DSS mice.

### Effects of Bovine Lactoferrin on Gut Microbial Metabolites in Dextran Sulfate Sodium Salt Mice

**Figure 4:**
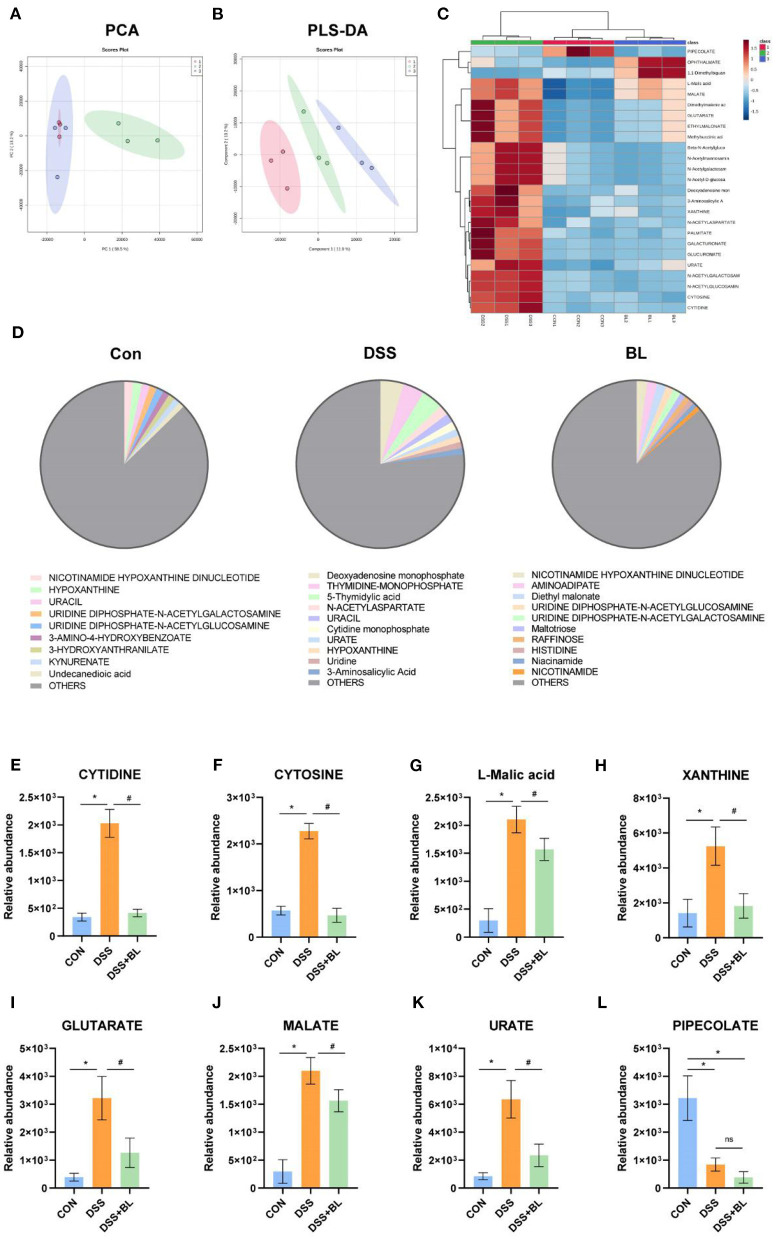
Effects of bovine lactoferrin (BL) on gut microbial metabolites in dextran sulfate sodium salt (DSS) mice. **(A)** Principal component analysis (PCA) of intestinal flora metabolites. **(B)** Partial least squares discriminant analysis (PLS-DA) of intestinal flora metabolites. **(C)** Top 25 common metabolites. **(D)** Proportion of top 10 metabolites in each group. **(E–L)** Relative abundance of top eight metabolites. One-way ANOVA was used to compare the three groups. *P < 0.05 vs. control; ^#^*P* < 0.05 vs. DSS.

In order to detect the effect of BL on the metabolic function of the intestinal flora in the DSS mice, we collected the metabolites of the flora in the feces of mice for metabonomic analysis. We utilized PCA and partial least squares discriminant analysis (PLS-DA) to analyze the data of metabolomics, although PCA results showed that the difference between the control group and the DSS+BL group was unapparent (Figure 4A). PLS-DA displayed that the metabolite composition was significantly different among the three groups (*P* < 0.05; Figure 4B). We analyzed the relative abundance of the top 25 metabolites (Figure 4C). Compared with control groups, in the DSS group, 22 metabolites were significantly increased (*P* < 0.05). The relative abundance of PIPECOLATE was the highest in the control group. In the DSS+BL groups, the relative abundance of OPHTHALMATE and 1,1-dimethylbiguanide was the highest. The other 22 metabolites, such as l-malic acid and MALATE, were highly expressed in the intestines of the DSS mice (*P* < 0.05). Next, we analyzed the top 10 metabolites of each groups (Figure 4D). We found that, in the control group and DSS+BL group, NICOTINAMIDE HYPOXANTHINE DINUCLEOTIDE had the highest proportion. In the DSS group, deoxyadenosine monophosphate had the largest proportion. We analyzed the relative abundance of the top eight metabolites in the three groups (Figures 4E–L). Except for PIPECOLATE, the expressions of the rest of the metabolites were up-regulated in the DSS group and decreased in the DSS+BL group. These results showed that BL participated in regulating the secretion of intestinal microbial metabolites flora in the DSS mice.

### Effects of Bovine Lactoferrin on Gut Microbial Structure in Dextran Sulfate Sodium Salt Mice

**Figure 5:**
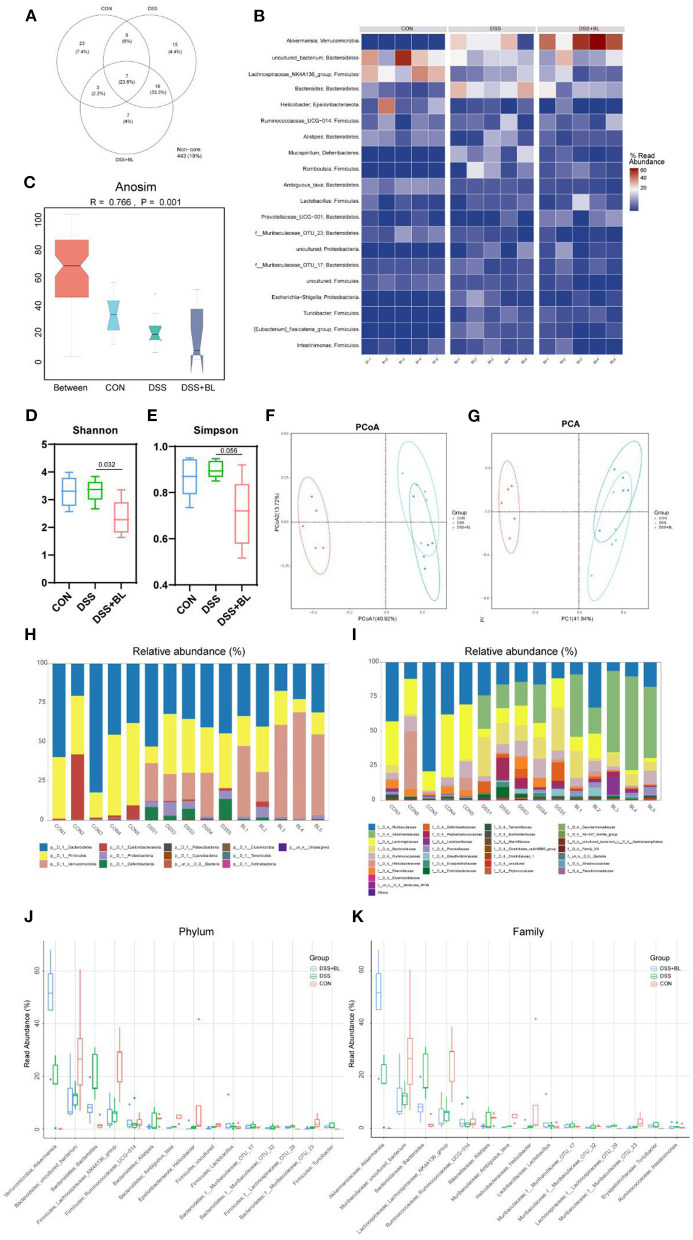
Effects of bovine lactoferrin (BL) on gut microbial structure in dextran sulfate sodium salt (DSS) mice. **(A)** Venn diagram of colony structure. **(B)** Top 20 intestinal flora in each group. **(C)** Anosim analysis. **(D)** Shannon analysis of intestinal flora. **(E)** Simpson analysis of intestinal flora. **(F)** Principal coordinate analysis (PCoA) of intestinal flora. **(G)** Principal component analysis (PCA) of intestinal flora. **(H,J)** The expression of phylum relative abundance. **(I,K)** The expression of family relative abundance. One-way ANOVA was used to compare the three groups. *P < 0.05 vs. control; ^#^*P* < 0.05 vs. DSS.

In order to detect the influence of BL on the structure and composition of the intestinal flora in the DSS mice, we collected the feces of three groups of mice and performed the sequence analysis of the microbes in the intestines for 16S rDNA sequencing to obtain the structure of the gut flora. In our results, three groups shared seven core bacterial groups in the Venn diagram (Figure 5A). The heat map showed that in the DSS+BL group, the expressions of *Akkermansia* were higher than those of the other groups (*P* < 0.05). The *uncultured_bacterium* and *Lachnospiraceae_NK4A136_group* were the lowest in the DSS groups compared with others (Figure 5B). The Anosim analysis showed that the difference between the groups of our results was greater than the difference within group (*P* = 0.001), and there was a significant difference (Figure 5C). The Shannon analysis showed that compared with that in the DSS group, the α-diversity of the gut flora in the DSS+BL group was significantly down-regulated (Figure 5D). However, in the Simpson analysis, there was no significant difference between the DSS group and DSS+BL group (Figure 5E). PCoA and PCA (β-diversity) showed that the genus difference between the DSS mice and normal mice was remarkable (Figures 5F,G). But the distance between the DSS group and the BL group is small and coincident. We analyzed the expressions of phylum (Figures 5H,J) and family (Figures 5I,K) relative abundance. In the phylum level, in the two colitis groups, Bacteroidetes and Firmicutes decreased, and Verrucomicrobia increased, compared with those in control group (*P* < 0.05). The proportion of Verrucomicrobia in the DSS+BL groups was higher than that in the DSS groups. In the family level, in colitis mice, Muribaculaceae and Lachnospiraceae decreased, and Akkermansiaceae increased, compared with that in the control group (*P* < 0.05). The proportion of Akkermansiaceae in the DSS+BL groups was higher than in the DSS groups. These results indicated that BL could change the structure and composition of the gut flora in the DSS mice, but the potential regulatory mechanism of BL on colitis requires our further exploration.

### Relationship Between Gut Microbial Diversity and Gut Microbial Metabolites

**Figure 6:**
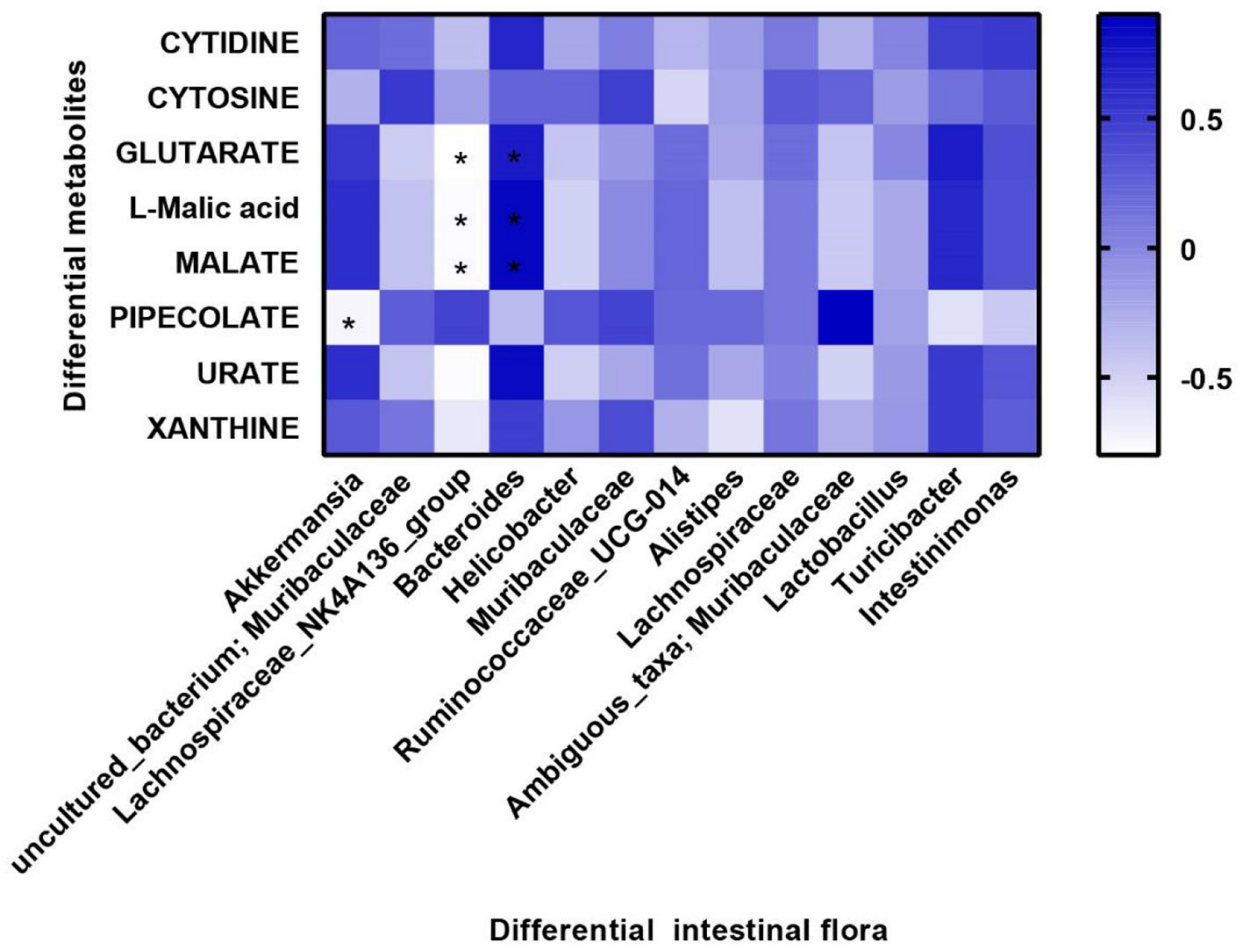
Correlation between microbial diversity and metabolites. Differential metabolites are displayed on the y-axis, and differential flora are displayed on the x-axis. Blue color represents positive correlation, and white color represents negative correlation. Spearman analysis was used to compare all groups. *P < 0.05 differential metabolites vs. differential intestinal flora.

Furthermore, we investigated the potential association between intestinal differential metabolites and intestinal microflora. We selected the top eight different intestinal metabolites and the top 15 different intestinal microflora at the family level of the three groups of mice for correlation analysis. The results showed that *Akkermansia* was negatively correlated with PIPECOLATE expression. *Lachnospiraceae_NK4A136* was negatively correlated with GLUTARATE, l-malic acid, and MALATE. However, *Bacteroides* was positively correlated with GLUTARATE, l-malic acid, and MALATE (Figure 6).

## Discussion

Colitis is a chronic inflammatory with hard recovery. BL is a kind of nutritional supplement that could repair the intestinal barrier function and intestinal microbiota to reduce enterohemorrhagic intestinal disease (22). Studies have shown that when BL was performed to treat colitis, the inflammatory response in the intestines was weakened and the barrier structure of the colon was protected (23). In our research, BL improved the damaged intestinal barrier in colitis and reduced inflammation in the colon. In addition, BL changed the intestinal microbes' structural diversity and metabolic function.

In our results, it was noted that BL alleviated the pathological symptoms of colitis and reduced body weight loss in colitis mice. We found that BL regulated the expression of colonic barrier defense-related proteins and tight junction proteins in colitis. A previous study found that the defense protein MUC2^−/−^ mice were suffering from malnutrition at 4 weeks old, and the abundance of the microbial community was more complicated than that of normal mice (24). Additionally, chitosan, another kind of nutritional supplement, attenuated the changes in colon tissue morphology and excessive inflammation caused by DSS, which was associated with increased ZO-1 expression (25). In this study, it was found that BL increased the expression of Claudin-1, Occludin, and ZO-1 in the intestines of colitis mice. In colitis, the epithelial barrier in the intestines was destroyed. The host's immune cells have increased contact with microorganisms in the intestinal tract, leading to frequent inflammatory reactions (26). Our results found that BL down-regulated the expressions of IL-1β, IL-6, and TNF-α inflammatory factors in the colon tissue of colitis mice and increased the expression levels of anti-inflammatory factors IL-10 and TGF-β. Therefore, we inferred that BL could be used as an auxiliary treatment to repair the colitis colonic barrier and reduce the inflammation in the colon.

It is well-known that intestinal microbes play a vital role in intestinal diseases. Gogokhia et al. showed that increasing bacteriophage levels could exacerbate colitis through TLR9 and IFN-γ (27). In our results, BL decreased the α-diversity of the flora, comparing with that in the DSS group. This means that BL inhibited the growth and reproduction of some intestinal flora in mice with colitis. The β-diversity showed that the distance between the DSS group and the BL group was coincident. This result showed that the types of flora in the BL group and the DSS group were similar. In our results, Muribaculaceae/Lachnospiraceae intestinal type in colitis mice with BL intervention turned into Akkermansiaceae/Bacteroidaceae intestinal type. Muribaculaceae acts a pivotal part in regulating the community composition and metabolites of microbial flora. Studies have shown that Muribaculaceae is a kind of bacteria beneficial to longevity in the intestinal flora (28, 29). Muribaculaceae participates in the degradation of polysaccharides, which will produce succinate, acetate, and propionate (30). These metabolites were beneficial to the intestinal barrier. Selective prebiotic-like effects on Akkermansiaceae also participated in the composition and metabolism of the flora and made the damaged intestines develop toward a healthy direction (31). However, the role of Lachnospiraceae and Bacteroidaceae for the host was still controversial (32). These results manifested that BL may change the structure of the excessively diverse intestinal flora in the DSS mice and made the composition of the flora move toward the direction of treating colitis. Meanwhile, BL reduced the expression of most of the metabolites of the intestinal flora, such as URATE. Excessive URATE could cause ventilation and kidney stones, which also made excessive inflammation in the intestines (33). Most of the metabolites that improved in the BL groups belong to purines. Therefore, we speculated that DSS colitis may cause a disorder of purine metabolism in the intestines. BL improved the structure of the intestinal flora, thereby restoring purine metabolism in the intestines. Tyson's research shows that *Saccharomyces cerevisiae* could treat colitis by improving purine metabolism (34). This experimental result shows that the intestinal flora of *Akkermansia* could decrease PIPECOLATE metabolite. *Lachnospiraceae_NK4A136* could decrease GLUTARATE, l-malic acid, and MALATE metabolites, while the effect of *Bacteroides* on the above three metabolites was opposite to that of *Lachnospiraceae_NK4A136*. In the future, we will do further experiments to specifically explore the effects of these intestinal flora and intestinal metabolites.

In the current study, we only found out that BL could regulate the gut flora in colitis mice, but the specific regulation mechanism of BL on intestinal flora and metabolism of mice with colitis still needs further research. In our next work, we will further explore this issue in order to clarify the mechanism of BL regulation of colitis.

## Conclusion

In conclusion, BL could relieve colitis mice through reducing the inflammatory reaction in colitis, protecting the intestinal barrier, and regulating the structural composition and metabolic function of intestinal microorganism. Hence, as an adjuvant therapy, BL may be clinically valuable.

## Data Availability Statement

The datasets presented in this study can be found in online repositories. The name of the repository is SRA and accession number is PRJNA699346.

## Ethics Statement

The animal study was reviewed and approved by Animal Ethics of the Second Xiangya Hospital, Central South University (2020844).

## Author Contributions

SW: conceptualization, validation, and formal analysis. JZ: methodology, formal analysis, and writing. DX: software, validation, and investigation. GS: formal analysis, investigation, and writing. LG: investigation, formal analysis, and writing. All authors: contributed to the article and approved the submitted version.

## Conflict of Interest

The authors declare that the research was conducted in the absence of any commercial or financial relationships that could be construed as a potential conflict of interest.
