# Supplementary material for: Bovine Lactoferrin Protects Dextran Sulfate Sodium Salt Mice Against Inflammation and Impairment of Colonic Epithelial Barrier by Regulating Gut Microbial Structure and Metabolites
Source: Front Nutr. 2021 Apr 16;8:660598. doi: 10.3389/fnut.2021.660598 (PMC8092122; doi:10.3389/fnut.2021.660598)
Supplement: Supplementary file 1 [file Presentation_1.PPTX]

## Slide 1
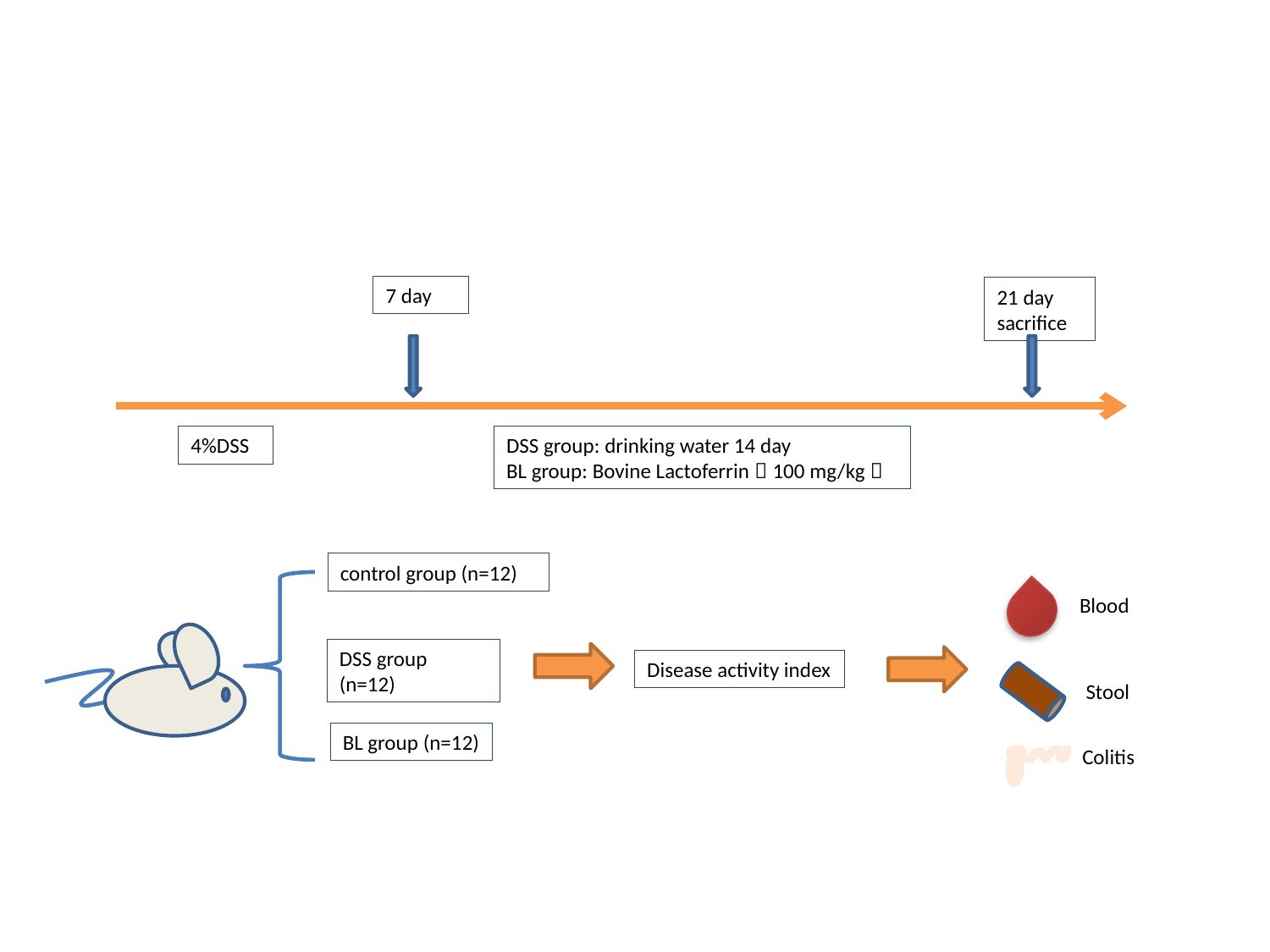

7 day
21 day
sacrifice
4%DSS
DSS group: drinking water 14 day
BL group: Bovine Lactoferrin（100 mg/kg）
control group (n=12)
Blood
DSS group (n=12)
Disease activity index
 Stool
BL group (n=12)
 Colitis
